# Single-Molecule Characterization of Cy3.5 -Cy5.5 Dye Pair for FRET Studies of Nucleic Acids and Nucleosomes

**DOI:** 10.1007/s10895-022-03093-z

**Published:** 2022-11-26

**Authors:** Mohamed Ghoneim, Catherine A. Musselman

**Affiliations:** Biochemistry and Molecular Genetics, University of Colorado Anschutz Medical Campus, 80045 Aurora, CO USA

**Keywords:** FRET, Single-molecule fluorescence microscopy, Cy3.5, Cy5.5, Double-stranded DNA, Nucleosome

## Abstract

Single molecule FRET (Forster resonance energy transfer) is very powerful method for studying biomolecular binding dynamics and conformational transitions. Only a few donor - acceptor dye pairs have been characterized for use in single-molecule FRET (smFRET) studies. Hence, introducing and characterizing additional FRET dye pairs is important in order to widen the scope of applications of single-molecule FRET in biomolecular studies. Here we characterize the properties of the Cy3.5 and Cy5.5 dye pair under FRET at the single-molecule level using naked double-stranded DNA (dsDNA) and the nucleosome. We show that this pair of dyes is photostable for ~ 5 min under continuous illumination. We also report Cy3.5-Cy5.5 FRET proximity dependence and stability in the presence of several biochemical buffers and photoprotective reagents in the context of double-stranded DNA. Finally, we demonstrate compatibility of the Cy3.5-Cy5.5 pair for smFRET in vitro studies of nucleosomes.

**Supplementary Information:**

The online version contains supplementary material available at 10.1007/s10895-022-03093-z.

## Introduction

Förster resonance energy transfer (FRET) is the non-radiative transfer of energy from a donor dye to an acceptor dye [1, 2]. This distance dependent transfer can be exceptionally useful for garnering proximity based information within and between molecules. It can be carried out in bulk or at the single-molecule level (smFRET). The latter provides insight into the activity of individual molecules and was first applied to the study of biomolecules in 1996 [3]. Over more than 25 years, this method has proven powerful in dissecting many aspects of inter-molecular interactions and intra-molecular conformational transitions of many types within biomolecular systems [2]. Importantly, when carried out at the single-molecule level, FRET provides insight into the heterogeneity between molecules of an ensemble [4, 5], including the ability to detect short-lived states [6, 7] and rarely visited states of biomolecules in real time. The most widely used approach to carrying out in vitro smFRET experiments is through labeling the biomolecules of interest with relatively small pairs of organic dyes [8]. Selection of a certain pair of dyes for smFRET studies is based on several criteria, including good separation of the emission of donor and acceptor dyes, similarity of the fluorescence quantum yields of the pair, commercial availability in multiple conjugation forms, and most importantly persistence of a stable emissive state for the longest time possible (i.e., long photobleaching time) and minimal occurrence of transient loss of emissivity (i.e., rare blinking) [9]. To date, the most commonly used pair of dyes in smFRET in vitro studies is the Cy3-Cy5 pair [8], with only a few other pairs of dyes (e.g., Alexa and Atto dyes) that are less frequently used. Hence, it is important to introduce and characterize new pair of dyes for smFRET biomolecular studies.

Here we focus our studies on the Cy3.5-Cy5.5 pair of dyes for FRET. These are related to the more commonly used Cy3-Cy5 pair, but with absorption and emission spectra that are shifted to longer wavelengths. The Cy3.5-Cy5.5 FRET pair have previously been used in ensemble biomolecular studies [10] and only very recently have been considered for smFRET studies [11]. Two recent single-molecule studies characterized the photophysical properties of the individual Cy3.5 and Cy5.5 dyes in absence of FRET [12, 13]. Here we characterized the properties of this pair of dyes at the single-molecule level under FRET while conjugated to either dsDNA or a nucleosome. We did this using total-internal reflection fluorescence microscopy (TIRFM); one of the most widely used types of microscopes for smFRET studies [2], and in the presence of the most routinely used types of photoprotective reagents [14, 15]. We demonstrate that this dye pair has stable emission for ~ 5 min, with rare blinking under FRET. We also provide the first single-molecule demonstration of proximity dependence of FRET between this pair of dyes while conjugated to dsDNA. Stability of FRET between this dye pair is also tested in the presence of several types of biochemical buffers and photoprotective reagents. Together, our results demonstrate that the Cy3.5-Cy5.5 pair is suitable for smFRET studies of DNA and chromatin substrates.

## Materials & Methods:

### Labeling and Annealing DNA Samples:

The complementary single-stranded DNA (ssDNA) oligonucleotides with internally amino-modified d-Thymine and a 6-carbon linker were purchased from Integrated DNA Technologies (IDT) with HPLC purification (see supporting material for DNA sequences). Cy3.5 and Cy5.5 NHS ester were purchased from AAT Bioquest (cat # 148 and 174, respectively). One of the ssDNA oligonucleotide pairs was biotinylated at 5’-end and labeled with Cy5.5 acceptor. The other ssDNA oligonucleotide was labeled with the Cy3.5 donor. For the dsDNA construct with 0-bp separation between dyes, the complementary ssDNA oligonucleotides were labeled differently (see supporting material for DNA sequences). One ssDNA oligonucleotide was biotinylated at 5’-end and labeled with Cy5.5 at 3’-end via amino-modified d-Thymine and a 6-carbon linker, while the other ssDNA oligonucleotide was labeled with Cy3.5 via 5’-amino-modifier with 6-carbon linker. Labeling and purification of ssDNA was carried out according to published protocols [9], with few modifications. Each labeling reaction was carried out in 80 µL total volume with 1 nmol ssDNA (12.5 μm) and 2.5 mM dye NHS easter. Reactions were incubated at 30 °C overnight with shaking. Labeled ssDNA were resuspended at 25 µM in 10 mM Tris-Cl (pH 8) then annealed according to published protocols [16], with final concentration of 1 µM dsDNA.

*Preparation of Doubly-labeled Fluorescent Nucleosomes*: The nucleosome was formed using human histones and the Widom 601 DNA. The Cy3.5-Cy5.5 pair was placed on the C-terminus of H2A and the entry/exit DNA. Human histones H2A K119C, H3 and H4 were expressed in Rosetta 2 (DE3) pLysS, while H2B was expressed in BL21 (DE3). They were purified according to previously published protocols [17]. The dsDNA used to reconstitute the nucleosome was designed such that it has a 6 bp linker on one side followed by the 147 bp Widom 601 sequence and a 100 bp linker on the other side. It was prepared by PCR and labeled with Cy5.5 and biotin at opposite ends using a pair of modified primers [18–20]. The 5’-amino modified (with 6-carbon linker) and 5’-biotinylated primers were purchased from IDT and the former was labeled with Cy5.5 NHS ester. The PCR product was purified using ethanol precipitation, non-denaturing PAGE, butanol extraction and a final ethanol precipitation [18]. H2A K119C was labeled with Cy3.5 maleimide and excess dye was removed using centrifugal filters (Microcon-5, MWCO 5 kDa, Biomax, Millipore) [19]. 16 nmole of fluorescent histone octamer was refolded using a molar ratio of 1.2:1 of H2A/H2B : H3/H4, respectively, by dialysis into high-salt buffer, then purified using size-exclusion chromatography (Superdex Increase 200, 10/300, Cytiva). Nucleosomes were reconstituted by mixing Cy5.5-labeled dsDNA with Cy3.5-labeled histone octamer at a 1.25:1 molar ratio, respectively, in high-salt buffer (2 M NaCl, 5 mM Tris-Cl, pH 8, 0.5 mM EDTA and 1 mM benzamidine) then exchanged to no-salt buffer (5 mM Tris-Cl, pH 8, 0.5 mM EDTA and 1 mM benzamidine) through three steps of double-dialysis. Nucleosomes were purified using ultra-centrifugation over 5–40% (w/v) sucrose gradient. Fractions were analyzed on 5% non-denaturing PAGE and fractions containing nucleosomes were pooled and concentrated to ~ 1 μM and stored at 4 °C in darkness.

### Single-molecule Fluorescence Imaging:

Images were collected using a Nikon Eclipse Ti objective -type total-internal reflection fluorescence microscope (TIRFM). Continuous wave diode-pumped solid state (DPSS) laser (561 nm; Coherent) is guided to an oil immersion 100X Objective lens via fiber optics to generate evanescent field of illumination for the excitation of Cy3.5. Fluorescence signals of Cy3.5 and Cy5.5 were collected by this same objective. Images were chromatically separated into Cy3.5 image and Cy5.5 image using 662 nm edge BrightLine dichroic mirror (Semroch, AVR Optics, cat. # FF662-FDI01) inside the dual view system (DV2; Photometrics). Scattered excitation light was removed in the emission optical pathway using Cy3.5 emission filter (Semroch, AVR Optics, cat. # FF01-612/69 − 25) and Cy5.5 emission filter (Semroch, AVR Optics, cat. # FF01-697/58 − 25) installed inside the dual view system. Images were recorded using EMCCD camera (Andor, DU-897E-C00-#BV). All dsDNA measurements were recorded at room temperature (21 ^o^C) using 100 ms/frame, except for data acquired to determine mean photobleaching times of Cy3.5 and Cy5.5 which was recorded with 250 ms/frame. Nucleosome data were recorded using 200 ms/frame. Slides and coverslips were cleaned and passivated according to published protocols [9, 21, 22]. Briefly, slides and coverslips were passivated using methoxy-PEG-SVA (MW = 5,000, Laysan Bio, Inc.) containing 2.5% biotin-PEG-SVA (Mr = 5,000, Laysan Bio, Inc.) in 100 mM sodium bicarbonate (pH 8.5). To immobilize biotinylated Cy3.5-Cy5.5-labeled dsDNA on slide surface, Neutravidin (0.2 mg/ml) in T50 buffer (10 mM Tris-HCl, pH 8, and 50 mM NaCl) was injected in imaging chamber and incubated for 5 min. Excess neutravidin was washed out with T50 buffer. Biotinylated dsDNA was prepared in buffer containing 25 mM Tris-Cl (pH 8), 100 mM NaCl and 0.1 mg/ml BSA and surface immobilized by incubation in the imaging chamber for 5 min. Excess dsDNA were washed and then immobilized dsDNA was imaged using imaging buffer containing 25 mM Tris-Cl (pH 8), 100 mM NaCl, 2 mM Trolox [14], and oxygen scavenging system (0.8% D-glucose, 2.17 U/uL catalase and 0.165 U/uL glucose oxidase) [9]. In case of testing compatibility of Cy3.5 and Cy5.5 with PCD/PCA oxygen-scavenging system, the imaging buffer was supplied with 2.5 mM PCA (ProtoCatechuic Acid) and 1.75 mU/uL (250 nM) PCD (ProtoCatechuate-3,4-Dioxygenase) enzyme [23]. In case of testing compatibility of Cy3.5 and Cy5.5 with different biochemical buffers, the imaging buffer contained glucose oxidase – catalase oxygen scavenging system supplied with 25 mM of the corresponding biochemical buffer tested. For nucleosomes, 0.2 mg/mL BSA in T50 buffer was injected into imaging chamber and incubated for 2 min. Excess BSA was washed with the same buffer, followed by injecting Neutravidin (50 ug/ml) in T50 buffer into imaging chamber and incubation for 5 min. Excess neutravidin was washed out with T50 buffer. Biotinylated nucleosomes were prepared in nucleosome buffer (25 mM Tris-Cl (pH 7.5), 100 mM KCl and 0.2 mg/ml BSA, 4% glycerol, 2 mM MgCl_2_, 1 mM DTT and 1 mM EDTA) and surface immobilized by injection then incubation in the imaging chamber for 5 min. Excess nucleosomes were washed with and then imaged in nucleosome buffer supplied with 2 mM Trolox and oxygen scavenging system (0.8% D-glucose 2.17 U/uL catalase and 0.165 U/uL glucose oxidase).

### Analysis of Single-molecule Data:

Extracting single-molecule intensity- time trajectories and FRET data from raw TIFF file videos were all done using MATLAB (MathWorks, Inc., R2019b version) [24]. Apparent FRET efficiencies were calculated as the ratio of acceptor intensity divided by the total of acceptor and donor intensities. For experiments with dsDNA, FRET data extracted by MATLAB were compiled into a time-binned FRET histogram and fit using IGOR Pro 9 version (WaveMetrics Inc.). Duration time data used for determining mean photobleaching time were obtained by visual inspection of single-molecule intensity-time trajectories in MATLAB, then compiled into 1 – CDF (1 - cumulative density function) and fit using IGOR Pro 9. For experiments with nucleosomes, the mean FRET efficiency of each of > 200 trajectories was determined from different experiments, then compiled into mean-FRET histogram and fit using IGOR Pro 9.

## Results & Discussion

### Photostability of Cy3.5 and Cy5.5 on dsDNA Under FRET:

**Fig. 1:**
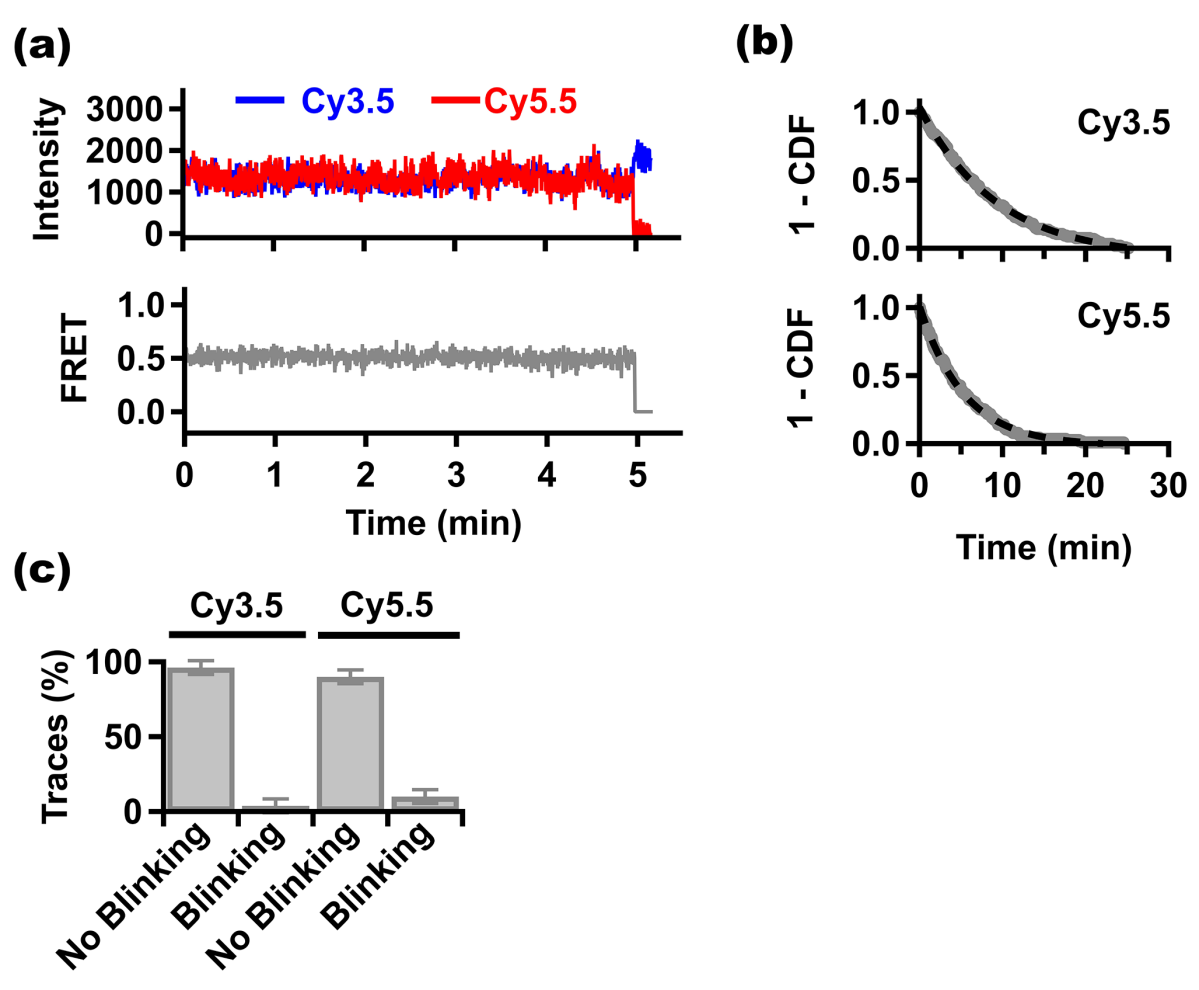
**Photostability of Cy3.5 and Cy5.5 dyes under FRET. (a)** Upper, representative single-molecule intensity – time trajectories of Cy3.5 (blue) and Cy5.5 (red). Lower, single-molecule FRET – time trajectory (gray). The single-molecule trajectories were recorded from surface-immobilized double-stranded DNA labeled with Cy3.5 and Cy5.5 that are separated by 16 bp. **(b)** 1 – CDF (or 1 – cumulative distribution function, gray line) of photobleaching time of Cy3.5 (upper, N = 162) and Cy5.5 (lower, N = 115) fit to a single exponential (black dashed line) under laser excitation power of 5 mW. **(c)** Percentages of Cy3.5 and Cy5.5 single-molecule trajectories that do not show blinking before the terminal photobleaching (N = 125). Error bars are ± standard deviation from multiple measurements

An ideal photostable dye for single-molecule fluorescence microscopy should have a long photobleaching time without frequent transient loss of emissivity (i.e., blinking) [25]. To assess this, we investigated Cy3.5 and Cy5.5 conjugated to double-stranded DNA (dsDNA) under FRET. Biotinylated dsDNA were labeled with Cy3.5 and Cy5.5 separated by 16 base pairs (bp) on opposite strands and immobilized on a neutravidin-coated surface according to routinely used methods [9, 22]. To assess photobleaching times of Cy3.5 and Cy5.5, we measured the intensity traces of isolated molecules in imaging buffer for 30 min under continuous 561 nm laser illumination with 5 mW. Representative single-molecule intensity- and FRET- time trajectories are shown in Fig. 1a. Time durations of > 100 individual trajectories of each dye were compiled into 1 – cumulative distribution then fit to a single exponential probability function to determine the mean photobleaching times of Cy3.5 and Cy5.5 which we found to be 9.3 ± 0.7 min and 5.4 ± 0.5 min, respectively (mean ± standard error. See Fig. 1b). This is slightly longer than the mean photobleaching times published for Cy3.5 and Cy5.5 conjugated to single-stranded DNA (ssDNA) [13], and also slightly longer than mean photobleaching times of the closely related Cy3 and Cy5 dyes that are most commonly used in the field of single-molecule fluorescence microscopy [23, 26]. It is important to note that all these previously published values of mean photobleaching time were determined for each dye alone, without FRET, while the values of the two mean times determined here for Cy3.5 and Cy5.5 were obtained under the effect of FRET. Indeed, we found that the mean photobleaching time of Cy3.5 alone conjugated to dsDNA of the same sequence is shorter in absence of FRET (5.38 ± 0.45 min. See supplementary Fig. S1). Blinking behavior of Cy3.5 and Cy5.5 dyes under effect of FRET was also assessed. Under the experimental conditions we used in this study, we determined that ~ 96% and ~ 90% of Cy3.5 and Cy5.5 single-molecule trajectories, respectively, do not show blinking within the time resolution of our measurements (100 ms/frame. See Fig. 1c). Due to paucity of traces associated with blinking, it is not possible here to provide accurate analysis of the blinking events of the two dyes. Roughly, in the minority of traces that are associated with blinking, there is ~ 1 and ~ 4 blinking events per Cy3.5 and Cy5.5 trajectory, respectively. The vast majority of these blinking events in this minority of trajectories last for 1–2 frames only, which means that blinking is not likely to cause any significant artifact in determining the value of FRET. Together these indicate that Cy3.5 and Cy5.5 are photostable under the effect of FRET.

**Fig. 2:**
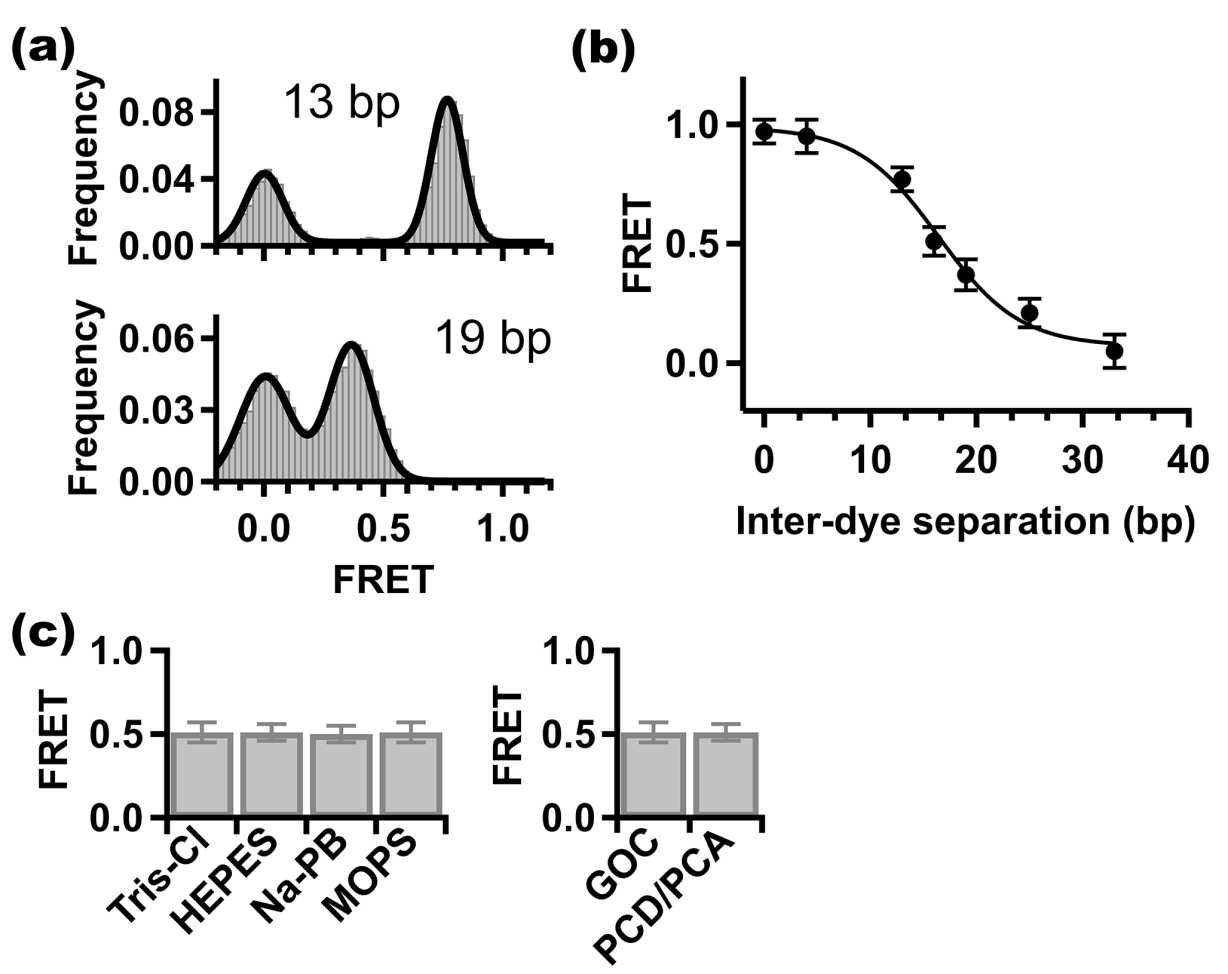
**Cy3.5-Cy5.5 FRET proximity dependence and stability in different buffers and photoprotective reagents. (a)** FRET histograms (gray bars) obtained from single-molecule trajectories for dsDNA doubly labeled with Cy3.5 and Cy5.5 separated by 13 bp (upper) and 19 bp (lower), respectively. Double gaussian fits (black line) provide the mean (and width) of the higher FRET peak. **(b)** Mean FRET efficiencies obtained from fitting FRET histograms plotted as a function of separation between Cy3.5 and Cy5.5 using different dsDNA constructs (N = 230, 227, 257, 230, 237, 164 and 163 for 0, 4, 13, 16, 19, 25 & 33 bp separations, respectively). The error bars are calculated from the spread of the FRET histograms. The sigmoidal line is only a visual guide. **(c)** Stability of mean FRET in different biochemical buffers (left) and photoprotective oxygen-scavenging systems (right). GOC is glucose oxidase – catalase system and PCD/PCA is Protocatechuate-3,4-dioxygenase - protocatechuic acid system. The error bars are calculated as in 'b' (N = 151, 118, 114, 120 and 105 for Tris-Cl, HEPES, Sodium phosphate (Na-PB), MOPS and PCD/PCA, respectively)

### Cy3.5-Cy5.5 FRET Proximity Dependence and Stability in Different Buffers and Photoprotective Reagents:

Knowing that the persistence length of B-form dsDNA is ~ 500 Å (~ 147 bp) under physiological-like conditions [27, 28], we decided to use it as a rigid spacer between fluorescent dyes in the distance range relevant to FRET (15–85 Å), an experimental approach adopted in the past in several single-molecule FRET studies [29], [31]. We prepared a set of doubly labeled dsDNA constructs with varying Cy3.5 (donor) – Cy5.5 (acceptor) separations to study the proximity dependence of this FRET pair on the single-molecule level. Based on the theoretically calculated Förster radius of Cy3.5-Cy5.5 pair (55 Å) [10], we studied dsDNA constructs with 0-, 4-, 13-, 16-, 19-, 25- and 33-bp separations between Cy3.5 and Cy5.5. Two examples of FRET histograms that were generated from single-molecule data for dsDNA with 13- and 19-bp Cy3.5-Cy5.5 separations are shown in Fig. 2a. Each of these histograms has two FRET peaks, one around zero, and another at higher FRET. The FRET peak around zero is commonly observed in a large number of single-molecule FRET studies [29],  [31], and it is suggested to be due to acceptor photobleaching, purification imperfections and a nonfluorescent (inactive) acceptor subpopulation. The second FRET peak shifts to lower FRET with increasing separation between dyes consistent with the expected FRET behavior [1, 32]. To determine the mean FRET value, we fit the FRET histograms to a double gaussian allowing for separation of the contribution of the higher FRET peak. A plot of mean FRET as a function of Cy3.5-Cy5.5 separation for all seven dsDNA constructs studied is shown in Fig. 2b. A monotonic decrease in FRET with Cy3.5-Cy5.5 separation is clearly observed. The solid sigmoid line in Fig. 2b is merely a visual guide as we do not attempt to fit the data to any model of dsDNA structure. It is important to note that in case of dsDNA construct with Cy3.5-Cy5.5 separation of 16 bp (roughly 54.4 Å), the measured mean FRET from our single-molecule FRET data is 0.51 ± 0.05, which suggests that the theoretically calculated Förster radius (55 Å) [10] is likely to be correct.

**Fig. 3:**
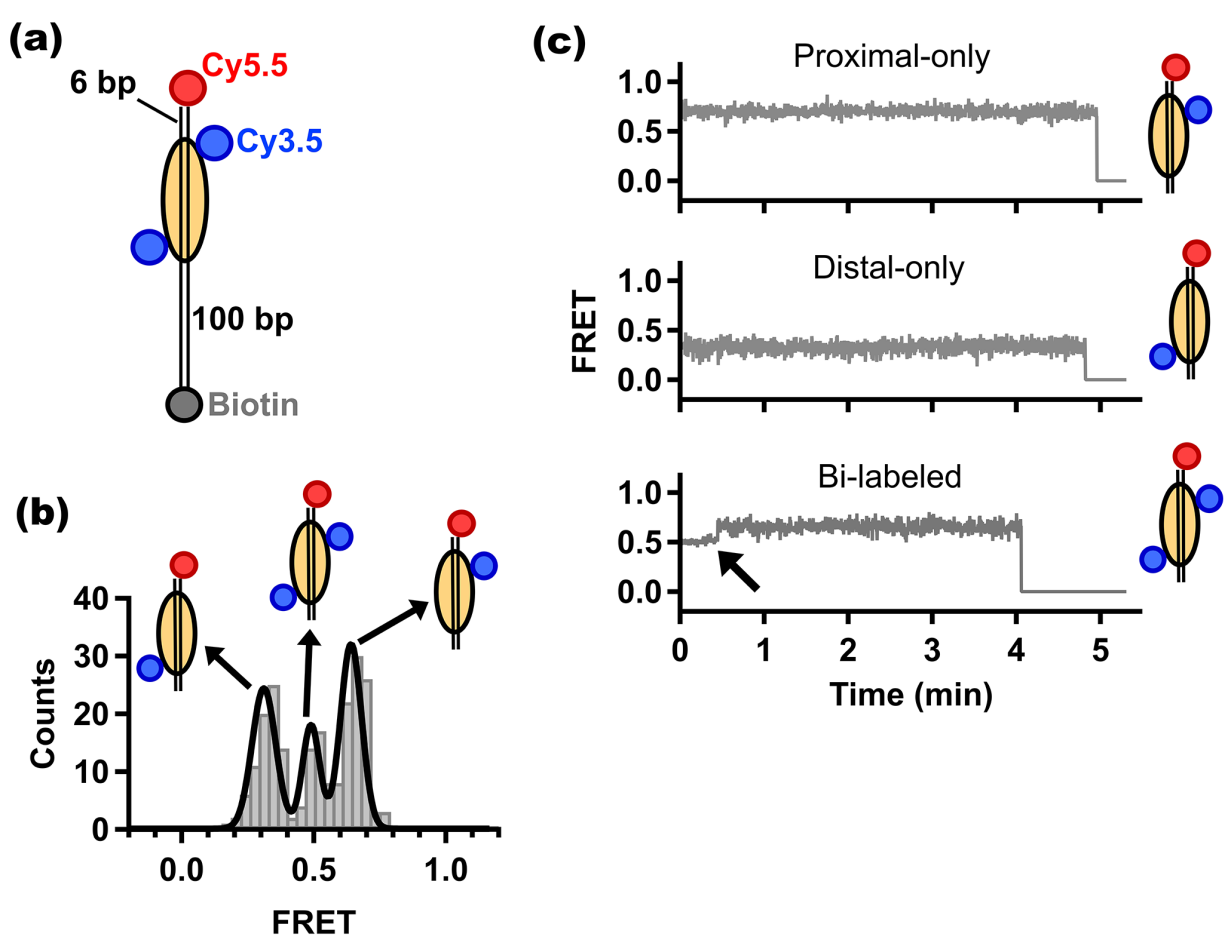
**Single-molecule FRET characterization of Cy3.5-Cy5.5 doubly-labeled fluorescent nucleosomes. (a)** Linear schematic diagram of doubly-labeled fluorescent nucleosome. Bi-labeled histone octamer (yellow oval) with Cy3.5 (blue) on each H2A is bound to double-stranded DNA (black lines) labeled with Cy5.5 (red) and biotin (gray) at opposite ends. The nucleosome is positioned 6 bp from the Cy5.5-labeled DNA end. The biotinylated end of dsDNA is 100 bp away from nucleosome. Biotinylated fluorescent nucleosome is immobilized on the neutravidin-coated surface of a microscope slide (not shown). **(b)** FRET distribution of the nucleosomes (gray bars). Three Cy3.5 labeling configurations results in three FRET peaks which Gaussian fit (black line) determined their centers to be at 0.64, 0.49 and 0.31 (N = 216). **(c)** Representative single-molecule FRET – time trajectories (gray) of proximal-only (upper), distal-only (middle) and bi-labeled (bottom) fluorescent nucleosomes. In all trajectories, FRET efficiency drops abruptly to zero due to photobleaching of one of the dyes. In the trajectory of bi-labeled nucleosome (bottom), the solid black arrow indicates photobleaching of distal donor first

Compatibility of Cy3.5-Cy5.5 FRET pair with different biochemical buffers and photoprotective reagents that are commonly used in single-molecule fluorescence studies has not been tested. Hence, we repeated our single-molecule FRET measurements for the dsDNA with 16 bp Cy3.5-Cy5.5 separation but in different biochemical buffers (25 mM Tris-Cl, pH 8, HEPES, pH 7.3, Na BP, pH 7.8, and MOPS, pH 7.5) and photoprotective reagents (GOC; Glucose Oxidase–Catalase and PCD/PCA; ProtoCatechuate-3,4-Dioxygenase - ProtoCatechuic Acid). Each of the photoprotective reagents tested work as an enzymatic oxygen-scavenging systems necessary for increasing the photobleaching time of fluorescent dye [25]. Figure 2c shows that the mean FRET value of single-molecule data obtained in all biochemical buffers and photoprotective reagents tested here is quite stable, which suggests that Cy3.5-Cy5.5 FRET pair is suitable for a broad spectrum of experimental conditions. Future investigations of the compatibility of this dye pair with more types of buffers will be necessary. Below, we demonstrate that this dye pair can be suitable for in vitro single-molecule studies of chromatin substrates.

### Single-molecule FRET Characterization of Cy3.5-Cy5.5 Doubly-labeled Nucleosomes:

The basic building block of chromatin is the nucleosome, which is key in regulation of the underlying genome. As such the structure, conformational dynamics, and binding of nucleosomes and nucleosome arrays is of major interest. smFRET studies of nucleosomes and nucleosome arrays have proven extremely valuable in understanding basic mechanistic principles of chromatin regulation. To demonstrate that the Cy3.5-Cy5.5 FRET pair is suitable for single-molecule in vitro chromatin studies, we doubly-labeled nucleosomes with this fluorescent pair. We generated nucleosomes containing Cy3.5-labeled H2A (at residue 119) and dsDNA labeled with Cy5.5 at one end and biotin at the other to assess the use of this pair [33] (Fig. 3a). The Widom 601 sequence [34] was used to position the octamer 6 bp away from the Cy5.5-labeled end of the dsDNA. Nucleosomes were separated from the biotinylated end by a 100 bp linker. Similar to experiments discussed in previous sections, biotinylated nucleosomes were immobilized on a neutravidin-coated microscope slide then imaged.

As each histone octamer has two H2A copies, fluorescent nucleosomes have multiple labeling configurations with Cy3.5 on H2A histone proximal to Cy5.5 (proximal-only labeled), or Cy3.5 on H2A histone distal to Cy5.5 (distal-only labeled), or Cy3.5 on both copies of H2A of the octamer (bi-labeled). Accordingly, we observed three peaks in the FRET distribution obtained from single-molecule FRET data of the doubly-labeled fluorescent nucleosomes (Fig. 3b). The three peaks are centered at FRET values of 0.64, 0.49 and 0.31 which correspond to proximal-only, bi-labeled and distal-only labeled nucleosomes, respectively. This pattern is consistent with that observed in previous studies of similarly labeled nucleosomes using different FRET pairs [22, 35, 36]. The proper assignment of the three FRET peaks to the different labeling configurations is evidenced from the number of photobleaching steps of the individual FRET-time trajectories. We can observe in Fig. 3c that proximal-only (upper) and distal-only (middle) labeled nucleosomes have FRET trajectories that photobleach with one step, while trajectory of bi-labeled nucleosome photobleach with two steps. In the example shown at the bottom of Fig. 3c, the nucleosome labeled with two Cy3.5 dyes had initial intermediate FRET (at ~ 0.5) and after the first photobleaching step (black solid arrow), FRET from single Cy3.5 on proximal H2A remained until the second photobleaching step.

The value of mean FRET of the proximal-only labeled nucleosomes is very similar to the corresponding value observed in single-molecule studies carried out using nucleosomes labeled at the same sites with the more commonly used Cy3-Cy5 FRET pair [37]. The theoretically calculated Förster radius of the Cy3.5-Cy5.5 pair [10] and the measured Förster radius of the Cy3-Cy5 pair [8] are similar, which suggests that FRET between each pair may depend on distance in a similar manner. In addition, both values of mean FRET determined here for proximal-only and distal-only labeled nucleosomes are consistent with a calibration of FRET-distance dependence that were carried out on nucleosomes doubly-labeled at the same sites, but using the AF555-AF647 FRET pair which also has a measured Förster radius [8] comparable to the theoretically calculated radius of Cy3.5-Cy5.5 pair [10]. Since it is hard to interpret single-molecule FRET data from two donors interacting with one acceptor, we will restrict our analyses in future studies to data from nucleosomes labeled with only one donor.

## Electronic Supplementary Material

Below is the link to the electronic supplementary material.

Supplementary Material 1

## Data Availability

Data available on request.
