# Supplementary material for: Single-Molecule Characterization of Cy3.5 -Cy5.5 Dye Pair for FRET Studies of Nucleic Acids and Nucleosomes
Source: J Fluoresc. 2022 Nov 26;33(2):413–21. doi: 10.1007/s10895-022-03093-z (PMC9957830; doi:10.1007/s10895-022-03093-z)
Supplement: Supplementary file 1 — Supplementary Material 1 [file 10895_2022_3093_MOESM1_ESM.pdf]

Supporting material

# Single-molecule characterization Cy3.5 - Cy5.5 dye pair for FRET studies of nucleic acids and nucleosomes

Mohamed Ghoneim[1*], Catherine A. Musselman[1]

[1] Biochemistry and Molecular Genetics, University of Colorado Anschutz Medical Campus, Aurora, CO, 80045, USA

** Corresponding author:*

mohamed.ghoneim@cuanschutz.edu (M.G.)

**Sequences of ssDNA oligonucleotides used to prepare Cy3.5-Cy5.5 doubly labeled dsDNA constructs:**

[A] For dsDNA constructs with 4-, 13-, 16-, 19-, 25-, & 33- bp separations between Cy3.5 and Cy5.5,

Biotinylated – **Cy5.5** labeled ssDNA sequence:

5'- /5BiotinTEG/AG GGA GCA CAC CGG T/iAmMC6T+**Cy5.5**/T GAA TAA ATG GAA ATA TAA AGT GAA ATG TCG TAT GAG GCG

Complementary **Cy3.5** labeled ssDNA sequences:

4 bp separation
5'- CGC CTC ATA CGA CAT TTC ACT TTA TAT TTC CAT TTA /iAmMC6T+**Cy3.5**/TC AAA CCG G
13 bp separation
5'- CGC CTC ATA CGA CAT TTC ACT TTA TAT /iAmMC6T+**Cy3.5**/TC CAT TTA TTC AAA CCG G
16 bp separation
5'- CGC CTC ATA CGA CAT TTC ACT TTA /iAmMC6T+ **Cy3.5**/AT TTC CAT TTA TTC AAA CCG G
19 bp separation
5'- CGC CTC ATA CGA CAT TTC ACT /iAmMC6T+ **Cy3.5**/TA TAT TTC CAT TTA TTC AAA CCG G
25 bp separation
5'- CGC CTC ATA CGA CAT /iAmMC6T+**Cy3.5**/TC ACT TTA TAT TTC CAT TTA TTC AAA CCG G
33 bp separation
5'- CGC CTC A/iAmMC6T+**Cy3.5**/A CGA CAT TTC ACT TTA TAT TTC CAT TTA TTC AAA CCG G

[B] For dsDNA construct with 0-bp separation between Cy3.5 and Cy5.5,

Biotinylated – **Cy5.5** labeled ssDNA sequence

5'- /5BiotinTEG/ AGG GAG CAC ACA TCG TCT GAT ACA TGG CTG TAA AGT ATG GAG ATT CTC TG/3AmMC6T+**Cy5.5**/

Complementary **Cy3.5** labeled ssDNA sequence

5'- /5AmMC6+**Cy3.5**/ CAG AGA ATC TCC ATA CTT TAC AGC CAT GTA TCA GAC GAT G

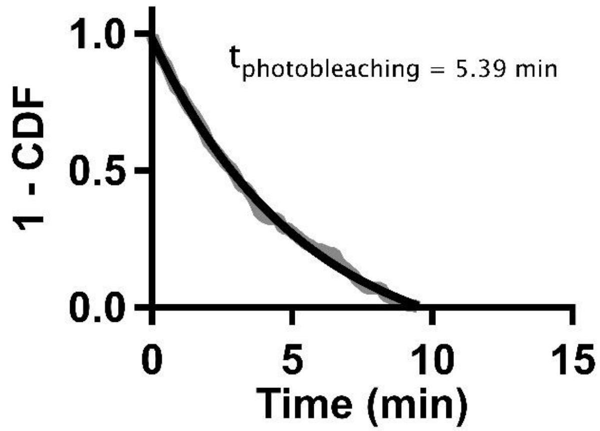


**Figure S1:**

1 – CDF (or 1 – cumulative distribution function), gray line, of photobleaching time of Cy3.5 alone (N = 142) fit to a single exponential (black line).
